# Diversity of mosquitoes and the aquatic insects associated with their oviposition sites along the Pacific coast of Mexico

**DOI:** 10.1186/1756-3305-7-41

**Published:** 2014-01-22

**Authors:** J Guillermo Bond, Mauricio Casas-Martínez, Humberto Quiroz-Martínez, Rodolfo Novelo-Gutiérrez, Carlos F Marina, Armando Ulloa, Arnoldo Orozco-Bonilla, Miguel Muñoz, Trevor Williams

**Affiliations:** 1 Centro Regional de Investigación en Salud Pública - INSP, Tapachula, Chiapas, Mexico; 2 Facultad de Ciencias Biológicas, Universidad Autónoma de Nuevo León, Nuevo León, Mexico; 3 Instituto de Ecología AC, Xalapa, Veracruz, Mexico

## Abstract

**Background:**

The abundance, richness and diversity of mosquitoes and aquatic insects associated with their oviposition sites were surveyed along eight states of the Pacific coast of Mexico. Diversity was estimated using the Shannon index (H’), similarity measures and cluster analysis.

**Methods:**

Oviposition sites were sampled during 2–3 months per year, over a three year period. Field collected larvae and pupae were reared and identified to species following adult emergence. Aquatic insects present at oviposition sites were also collected, counted and identified to species or genus.

**Results:**

In total, 15 genera and 74 species of mosquitoes were identified: *Anopheles pseudopunctipennis*, *An. albimanus* and *Aedes aegypti* were the most abundant and widely-distributed species, representing 47% of total mosquito individuals sampled. New species records for certain states are reported. Anopheline diversity was lowest in Sinaloa state (H’ = 0.54) and highest in Chiapas (H’ = 1.61) and Michoacán (H’ = 1.56), whereas culicid diversity was lowest in Michoacán (H’ = 1.93), Colima (H’ = 1.95), Sinaloa (H’ = 1.99) and Jalisco (H’ = 2.01) and highest in Chiapas (H’ = 2.66). In total, 10 orders, 57 families, 166 genera and 247 species of aquatic insects were identified in samples. Aquatic insect diversity was highest in Chiapas, Oaxaca and Michoacán (H’ = 3.60-3.75). Mosquito larval/pupal abundance was not correlated with that of predatory Coleoptera and Hemiptera.

**Conclusion:**

This represents the first update on the diversity and geographic distribution of the mosquitoes and aquatic insects of Mexico in over five decades. This information has been cataloged in Mexico’s National Biodiversity Information System (SNIB-CONABIO) for public inspection.

## Background

Vector-borne diseases transmitted by mosquitoes of the family Culicidae are responsible for ~1.4 million deaths per year [1] and 17% of all infectious diseases worldwide [2]. The principal pathogens transmitted by these vectors include viruses (dengue, yellow fever, equine encephalitis, etc.), protozoa (e.g., those causing malaria), and nematodes (e.g. those causing filariasis) [3]. Overall, fewer than 150 species of the genera *Anopheles*, *Aedes* and *Culex*, are the indirect cause of morbidity and mortality among humans, more than any other group of organisms [4].

To date, between 18 and 20 genera and 225–247 species of mosquitoes have been reported from Mexico [5-7]. However, only the subfamilies Anophelinae and Culicinae include vector species of medical or veterinary importance, especially those from the genera: *Aede*s, *Anopheles*, *Culex*, *Haemagogus*, *Mansonia*, *Sabethes*, *Psorophora*, and *Coquillettidia*[8,9]. These species oviposit in a wide range of aquatic habitats that also harbor numerous species of aquatic insects and plant species with which they interact. Species interactions are central to the ecology of any habitat, including those of mosquitoes [10]. Identification of the habitats selected as oviposition sites is of clear relevance to mosquito surveillance programs as these habitats are also targeted by mosquito control measures involving habitat elimination or larviciding activities [11].

Studies on the bionomics of major mosquito vectors in Mexico have been made [12-16], but few studies have addressed their interactions with other organisms, particularly those involving aquatic insects associated with their oviposition sites [17-21]. Overall 13 orders of insects include species with aquatic or semi-aquatic stages, representing >95% of macroinvertebrate species present in aquatic habitats [22,23]. Aquatic insects play key roles in the ecology of aquatic ecosystems and together with other invertebrates, exert an important influence on nutrient cycles and the structure of trophic webs [24]. Apart from their use as biological indicators to evaluate water quality [25], aquatic insects, particularly predatory insects, can play an important role in the biological control of larval and pupal mosquito populations [26].

In this study we describe a comprehensive analysis on the richness, diversity and geographical distribution of mosquitoes in Mexico and the aquatic insects associated with their oviposition sites. The entomological surveys in this study were restricted to the oviposition and immature development sites of mosquitoes because information on the diversity and distribution of endemic vector species is essential to develop vector monitoring and control strategies, which depend on the identity of mosquito species present in each state for effective implementation. This is because public health programs aimed at vector control are decided on a state-by-state basis, depending on state administration budgets and the perceived importance of mosquito control measures and vector borne diseases in each of the 31 states of Mexico. Similarly, this type of baseline information allows detection of changes in the distribution or abundance of species and detection of introduced species of vectors that have extended beyond their natural distribution or biogeographic areas (termed invasive species), and which can cause environmental, economic, and human health impacts.

## Methods

### Study area

**Figure 1:**
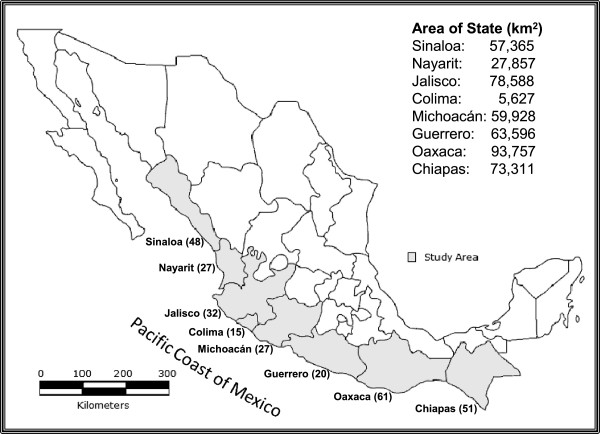
**States of the Pacific coast of Mexico included in the study.** Values in parentheses indicate total number of samples taken from each state. The surface area (km^2^) of each state is given.

The study area comprised eight states of the Pacific coast of Mexico, namely Sinaloa, Nayarit, Jalisco, Colima, Michoacan, Guerrero, Oaxaca and Chiapas (Figure 1). The climatic conditions along the Pacific coast region were predominantly characterized by a warm humid climate, with average annual temperature between 22° and 26°C and an annual rainfall of 1,000 to 2,000 mm [27]. The experimental design consisted of three rounds over the eight study states, one per year for about 55 days, the first was from October 13 to December 6, 2007, the second from September 20 to November 13, 2008 and the third from May 30 to July 23, 2009, giving a total of 165 days of sampling. The selection of sampling sites was based on the reports of type localities for native species of mosquitoes [5,8], and available information on the geographical distribution of mosquito species of medical and veterinary importance in the country [6,17,28-30]. In each state, temporary and permanent aquatic sites were sampled that represent potential habitats for the development of larval and pupal populations of mosquitoes, such as pools, lakes, streams, rivers, canals, marshes, etc. In addition, visits were made to cemeteries to collect immature stages of mosquitoes in water tanks. The sampling effort varied from 15 to 61 sites per state (Figure 1). Based on area, the most intensively sampled state was Colima with an average of 2.6 samples per 1000 km^2^, the least sampled state was Guerrero with an average of 0.3 samples per 1000 km^2^, whereas the average of all states was 0.6 samples per 1000 km^2^.

### Sampling methods and identification

Mosquitoes recorded in this study were obtained from field collections, and were compared to information from bibliographic records reported in scientific journals for different states in the study area. Larvae and pupae of mosquitoes were collected by using white enameled dippers, (500 ml capacity), with a flat outer side. All the larvae and pupae from each aquatic site were collected, counted, placed in 8 oz plastic sample bags, transported to the laboratory and then transferred to 1.5 mL plastic vials for adult emergence. Mosquitoes that emerged were placed in perforated plastic vials and were stored in plastic containers with silica gel for transport to the laboratories of the Regional Center for Public Health Research, National Public Health Institute (CRISP-INSP), Tapachula, Chiapas. Adult mosquitoes were identified to species using dichotomous keys [31-33].

Aquatic insects were collected using an aquatic entomological net (24 × 46 cm and mesh size 0.9 mm) that was dragged across the bottom and the surface of the water body at each aquatic site. In the case of lakes, rivers and streams an area of 5 m^2^ was sampled for 5 minutes using the traveling kick method [34]. Aquatic insect samples were sorted in white metal trays and were then preserved in 96% ethanol, taken to the laboratory, and identified to genus and species using the appropriate keys [22,35-42]. Reference specimens of all mosquitoes and aquatic insects were deposited in the entomological collection of CRISP-INSP. Field collection records were entered into the CONABIO Biótica information system 4.5 [43], to create a biodiversity database.

### Data analysis

The abundance of aquatic insects recorded in each state were subjected to multivariate analysis of variance (MANOVA) following *ln (x + 1)* transformation to normalize the distribution and eliminate zero values. For each of the eight states, α diversity of mosquitoes and aquatic insects was estimated by means of the Shannon index (H’) [44]. A randomization test was applied to determine significant differences between H’ values using the Species Diversity and Richness III (v. 3.0.2) program [45], which is based on the method described by Solow [46]. The accuracy of index values was estimated by jackknifing, which permitted a reduction in the bias in our estimate of the population value and provided a standard error [47]. Confidence intervals for the statistic were calculated by bootstrap with replacement. Differences amongst samples and the communities of mosquitoes and aquatic insects of all the states (β diversity), were estimated by calculating a quantitative similarity index (Morisita-Horn) and cluster analysis [44,48]. Correlations of the number of mosquitoes sampled against certain orders of aquatic insects order was performed by Spearman rank correlation in Statistica v.7 (StatSoft Inc. Tulsa, OK).

## Results

### Abundance and species richness

**Figure 2:**
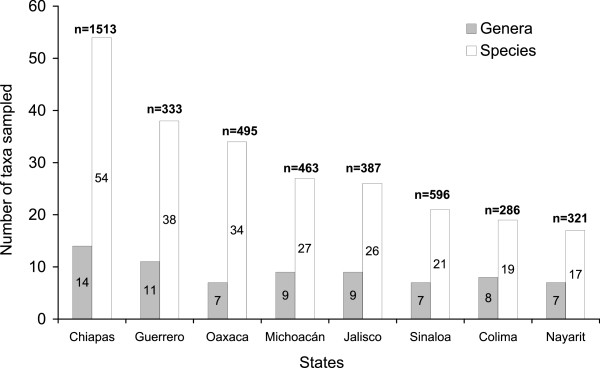
**Genera and species of mosquitoes from eight states of the Pacific coast of Mexico.** Numbers within columns indicate number of taxa. Numbers above columns indicate number of individuals identified.

Overall, sampling resulted in the identification of mosquitoes representing 15 genera, 74 species and 4394 individuals distributed among the eight states of the Pacific coast of Mexico. Of the mosquitoes identified to species, 23% of individuals were from seasonal rain pools followed by streams (22%), river margins (18%), lakes and lagoons (14%), marshes (10%), irrigation channels (9%), water tanks (2%), dams (1%) and sewers (1%). At the state level, Chiapas had the highest abundance and richness of culicid taxa, with 1513 individuals (Figure 2), distributed among 14 genera and 54 species, followed by Guerrero and Oaxaca with an abundance of 333 and 495 individuals, respectively, and a richness of 11 and 7 genera, and 38 and 34 species, respectively. The states of Michoacán, Jalisco and Sinaloa had intermediate abundance and richness of mosquitoes that fluctuated between 387–596 individuals from 7 to 9 genera and 21 to 27 species. The states with the lowest abundance and richness of taxa were Colima and Nayarit, with abundance and richness values less than half those observed in Chiapas (Figure 2).

Overall, the most abundant mosquito species were *Anopheles pseudopunctipennis*, *Aedes aegypti* and *An. albimanus*, with 747 (17.0%), 664 (15.1%) and 646 (14.7%) individuals sampled, respectively (Additional file 1: Table S1). These species represented 47% of the total mosquitoes collected and were recorded in all eight states of the study area. Additional species that were common and widely distributed in the eight states were *Ochlerotatus taeniorhynchus, Culex quinquefasciatus, Cx. coronator*, and *Cx. nigripalpus,* which together represented >20% of the specimens collected. In contrast, the distribution of a number of rare species was restricted to a single state. For example, *Ae. albopictus, Ae. angustivitatus*, *An. darlingi, An. gabaldoni*, *An. neivai*, *An. neomaculipalpus*, *An. vestitipennis*, *Coquillettidia venesuelensis*, and *Co. nigricans* were collected only in Chiapas. Similarly, *Och. infirmatus* was only identified in Guerrero, *Och. hastatus* was collected only in Colima, *Psorophora mathesoni* was restricted to Jalisco, and *Uranotenia orthodoxa* was identified only in samples from Michoacán (Additional file 1: Table S1). Overall, *Anopheles* mosquitoes were the most abundant with 1749 individuals and 19 species, representing almost 40% of the identified mosquitoes, followed by the genus *Culex* with 909 individuals (21%) and 12 species, and *Ochlerotatus* with 618 individuals (14%) and 9 species. The remaining 25% (1118 individuals) included 18 genera and 34 additional species of mosquitoes (Additional file 1: Table S1).

Due to their importance in the transmission of malaria, anopheline mosquitoes were considered to be of particular interest. In total, 18 species of anophelines were recorded during the development of this study (Table 1), and the state of Chiapas had the highest abundance and richness, with 941 individuals, and 15 species, followed by Michoacán and Oaxaca with 48 and 216 individuals, and 9 and 8 species, respectively. Only 5 species were registered in the states of Guerrero, Jalisco and Nayarit with 170, 70 and 95 individuals, respectively. The states with the least species richness were Colima and Sinaloa with 3 species, and 55 and 154 individuals, respectively (Table 1; Additional file 1: Table S1).

**Table 1:** Mosquito species recorded from eight states of the Pacific coast of Mexico in previous studies and the present study

**Species**	**Sinaloa**	**Nayarit**	**Jalisco**	**Colima**	**Michoacán**	**Guerrero**	**Oaxaca**	**Chiapas**
**(A) Culicids**								
*Aedes aegypti*	x*	x*	x*	x*	x*	x*	x*	x*
*Aedes albopictus*								*
*Aedes angustivittatus*								*
*Aedes atropalpus*				**x**	**x**	**x**	**x**	**x**
*Aedes knabi*							**x**	
*Aedes quadrivittatus*							**x**	**x**
*Aedes sexlineatus*								**x**
*Aedes vexans*			x*		x*			
*Aedes terrens*			x*		x*	‡*	x*	x*
*Aedeomyia squamipennis*						x‡*		*
*Coquillettidia nigricans*								*
*Coquillettidia venezuelensis*								*
*Culex apicalis*							**x**	
*Culex arizonensis*								**x**
*Culex bidens*	**x**							**x**
*Culex bigoti*					**x**	**x**	**x**	**x**
*Culex bihaicolus*								**x**
*Culex chidesteri*						x‡*	*	
*Culex conspirator*	x*					‡*	x*	x*
*Culex corniger*	x*					‡*	x*	x*
*Culex coronator*	x*	*	x*	x*	x*	x‡*	x*	x*
*Culex declarator*						**‡**		
*Culex derivator*								**x**
*Culex educator*						**‡**		
*Culex elevator*								**x**
*Culex erraticus*	x*	*	*	*	*	x‡*	x*	*
*Culex herythrothorax*					**x**			
*Culex inflictus*				**x**				
*Culex inpatiens*								**x**
*Culex interrogator*	x*				x*	*	x*	x*
*Culex iolambdis*			x*			‡*	x*	x*
*Culex maccrackenae*							**x**	**x**
*Culex mutator*								**x**
*Culex nigripalpus*	x*	x*	x*			x‡*	x*	x*
*Culex peccator*						**‡**		
*Culex pilosus*								**x**
*Culex pinarocampa*						**x**	**x**	
*Culex quinquefasciatus*	x*	*	*	x*	*	x‡*	x*	x*
*Culex restrictor*						*	x*	x*
*Culex restuans*					**x**			
*Culex salinarius*							**x**	
*Culex stigmatosoma*	x*	*	x*	*	x*	x*	x*	x*
*Culex taeniopus*						**‡**		
*Culex tarsalis*	**x**					**x**		**x**
*Culex thriambus*	x*				**x**	‡*	**x**	**x**
*Culex trifidus*						**x**		
*Culex virgultus*					**x**	**‡**	**x**	**x**
*Culiseta particeps*					*			
*Deinocerites belkini*			*			‡*		
*Deinocerites epitedeus*	**x**	**x**						
*Deinocerites howardii*			*					*
*Deinocerites pseudes*			x*	*		x‡*		
*Haemagogus equinus*			x*	*	x*	x‡*	x*	x*
*Haemagogus mesodentatus*								**x**
*Limatus durhamii*								x*
*Mansonia dyari*		*	*					
*Mansonia indubitans*							x*	x*
*Mansonia nigricans*						‡*		
*Mansonia titillans*	*	**x**		**x**		x‡*		x*
*Mansonia venezuelensis*								**x**
*Ochlerotatus angustivittatus*						‡*	x*	x*
*Ochlerotatus atropalpus*				*	*	‡*	*	*
*Ochlerotatus euplocamus*							**x**	
*Ochlerotatus epactius*	*	*	*	*	*			*
*Ochlerotatus infirmatus*						‡*		
*Ochlerotatus hastatus*				x*				
*Ochlerotatus nigromacul*						**x**		
*Ochlerotatus podographicus*		*						*
*Ochlerotatus scapularis*			*			x*		x*
*Ochlerotatus serratus*			**x**					**x**
*Ochlerotatus shannoni*					**x**			
*Ochlerotatus stigmaticus*							**x**	
*Ochlerotatus taeniorhynchus*	x*	x*	x*	x*	x*	x‡*	x*	x*
*Ochlerotatus thelcter*	**x**							
*Ochlerotatus trivittatus*					x*	**x‡**	x*	
*Ochlerotatus torilis*						**x**		
*Psorophora champerico*	**x**							**x**
*Psorophora ciliata*							**x**	**x**
*Psorophora cilipes*						**‡**		
*Psorophora confinnis*	x*				**x**		x*	x*
*Psorophora cyanescens*	x*		x*			‡*	x*	x*
*Psorophora discolor*							x*	x*
*Psorophora ferox*					x*	‡*	x*	x*
*Psorophora howardii*						**x**		**x**
*Psorophora lutzii*						‡*	x*	x*
*Psorophora mexicana*							**x**	
*Psorophora mathesoni*			*					
*Psorophora varipes*			x*	x*		‡*	x*	*
*Psorophora virescens*		x*		x*		x*	x*	
*Sabethes chloropterus*					x*			x*
*Uranotaenia coatzacoalcos*					**x**		**x**	**x**
*Uranotaenia geometrica*								**x**
*Uranotaenia lowii*	x*	*	*	*		‡*		*
*Uranotaenia orthodoxa*					*			
*Uranotaenia pulcherrima*						**x**		
*Uranotaenia socialis*	*			*				
*Uranotaenia sapphirina*	x*			x*	x*	x‡*		x*
*Wyeomyia arthrostigma*								**x**
*Wyeomyia celaenocephala*								**x**
*Wyeomyia jocosa*								**x**
*Wyeomyia mitchelli*								x*
*Wyeomyia personata*						‡*		x*
**(B) Anophelines**								
*Anopheles albimanus*	x*	x*	x*	x*	x*	x‡*	x*	x*
*Anopheles apicimacula*					x*			x*
*Anopheles argyritarsis*		x*			x*		x*	x*
*Anopheles aztecus*			x*		x*			
*Anopheles crucians*				*		‡*	*	*
*Anopheles darlingi*								x*
*Anopheles eiseni*					x*	x*	x*	x*
*Anopheles franciscanus*			*				*	*
*Anopheles freeborni*					*			
*Anopheles gabaldoni*								x*
*Anopheles hectoris*								x*
*Anopheles neivai*								x*
*Anopheles neomaculipalpus*								x*
*Anopheles parapunctipennis*							x*	x*
*Anopheles pseudopunctipennis*	x*	x*	x*	x*	x*	x‡*	x*	x*
*Anopheles punctimacula*	*	x*			x*	x‡*	x*	x*
*Anopheles punctipennis*		*	*		x*			
*Anopheles vestitipennis*								x*
*Anopheles xelajuensis*							**x**	
**Species records**	**Sinaloa**	**Nayarit**	**Jalisco**	**Colima**	**Michoacán**	**Guerrero**	**Oaxaca**	**Chiapas**
No. species from previous records (**x**) [28,29]	22	10	16	13	29	52 ‡	46	67
No. species in present study (*)	21	17	26	19	27	38	34	54
Previous species records confirmed in present study	17	8	15	10	20	36	30	40
Species exclusive to this study	4	9	11	9	7	2	4	14
Total number of species per state	26	19	27	22	36	54	50	81

**x**Indicates species reported in previous studies [28,29].

*Indicates species reported in the present study.

**‡**Double dagger symbol indicates species records from Guerrero state [17].

### Abundance and taxa richness of aquatic insects

**Figure 3:**
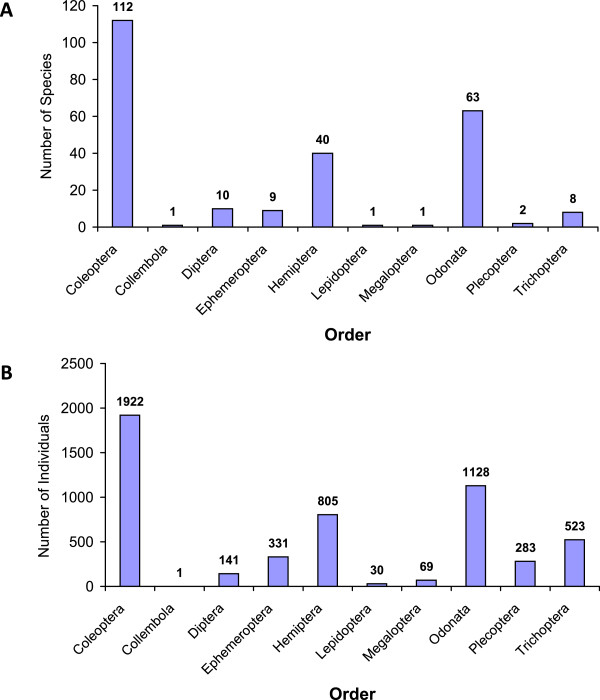
Numbers of (A) species and (B) individuals identified from 10 orders of aquatic insects sampled from mosquito oviposition sites in eight states of the Pacific coast of Mexico.

**Figure 4:**
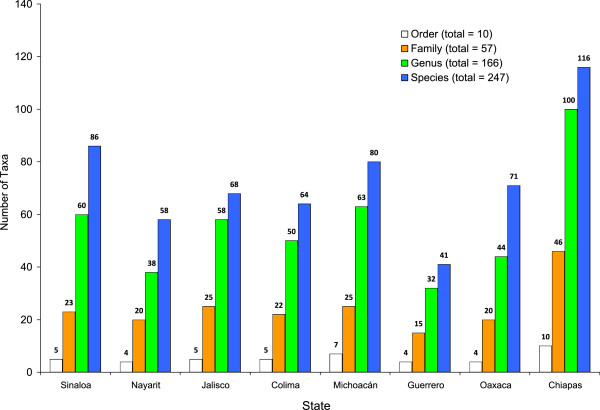
Taxa richness of aquatic insects sampled from mosquito oviposition sites in eight states of the Pacific coast of Mexico.

A total of 5233 individuals of aquatic insects were collected, identified and assigned to a total of 10 orders, 57 families, 166 genera and 247 species (Table 2). Significant differences were detected in the abundance of species between collections made in different states along the Pacific coast (MANOVA, Pillai’s Trace: *F*_8,240_ = 7.683; *P* < 0.0001). The order Coleoptera had the highest abundance of aquatic insects with 1922 individuals and 112 species, followed by the orders Odonata and Hemiptera, with 1128 and 805 individuals, and 64 and 40 species, respectively. Trichoptera, Ephemeroptera, Plecoptera and Diptera (not including mosquitoes) presented an intermediate abundance with 523, 331, 283 and 141 individuals, and 8, 9, 2 and 10 species, respectively. The least represented groups in the collections were Collembola with only one individual and one species, and Lepidoptera and Megaloptera, with 30 and 69 individuals respectively, each represented by a single species (Figure 3A and 3B). Chiapas state had the highest taxa richness, with 116 species distributed in 100 genera, 46 families and 10 orders (Figure 4), followed by the states of Sinaloa, Michoacan and Oaxaca, with between 86 and 71 species. The states with the lowest species richness were Jalisco, Colima, Nayarit and Guerrero with between 68 and 41 species collected during the sampling program (Figure 4).

**Table 2:** Aquatic insect species associated with the oviposition sites of mosquitoes and their presence in each of eight states* along the Pacific coast of Mexico

**Species**	**Distribution**	**Species**	**Distribution**	**Species**	**Distribution**	**Species**	**Distribution**	**Species**	**Distribution**
*Abedus ovatus*	Ch, G, O	*Crenitis* sp.	C, M	*Hesperagrion heterodoxum*	O	*Macrelmis* sp.	Ch	*Phyllogomphoides* sp.	Ch
*Ablabesmyia* sp.	C	*Cryphocricos* sp.	Ch	*Hesperocorixia vulgaris*	Ch	*Macronychus* sp.	Ch	*Phyllogomphoides suasus*	Ch, G, N
*Acilius* sp.	J, M, N	*Curicta howardi*	O	*Hetaerina cruentata*	Ch, G, N, O	*Macrothemis inacuta*	G, N, O	*Platyvelia* sp.	Ch
*Acneus* sp.	Ch	*Curicta* sp.	J, S	*Hetaerina vulnerata*	J, N, S	*Macrothemis pseudimitans*	Ch, G, N, O, S	*Potamyia flava*	Ch
*Rhionaeschna multicolor*	J, M	*Cybister* sp.	C, G, J, M, N, O, S	*Hetarina americana*	G, J, N, O	*Macrothemis ultima*	J, S	*Progomphus clendoni*	Ch
*Agabinus* sp.	J	*Cylloepus* sp.	Ch	*Heteragrion tricellulare*	Ch, O	*Macrovelia hornii*	Ch	*Progomphus* sp.	Ch
*Agabus* sp.	Ch	*Cyphon* sp.	J, N, S	*Heterelmis obesa*	Ch, O	*Macrovelia* sp.	M	*Psephenus* sp.	Ch
*Ambrysus mormon*	C, Ch, J, S	*Derallus rudis*	C, Ch, O, S	*Hydaticus* sp.	C, Ch, J, M, S	*Megadytes* sp.	J, M	*Pseudoleon superbus*	J, M, S.
*Ambrysus* sp.	C, G, M, N, O, S	*Derovatellus* sp.	S	*Hydraena* sp.	J	*Mesovelia mulsanti*	C, N	*Ranatra* sp.	C, Ch, G, J, M, N, O, S
*Anacaena suturalis*	C, J, M, S	*Desmopachria* sp.	C, J, M, N	*Hydrobiomorpha casta*	J. M, O, S	*Mesovelia* sp.	Ch, C, J, M	*Rhagovelia* sp.	C, Ch, O
*Anacroneuria* sp.	Ch	*Desmopachria striola*	S	*Hydrocanthus oblongus*	J, N	*Metrobates* sp.	Ch	*Rhantus calidus*	O
*Anax amazili*	C, Ch, N, S	*Dicranopselaphus* sp.	Ch	*Hydrocanthus* sp.	C, G, J, M, N, O, S	*Micrathyria aequalis*	Ch, N, J	*Rhantus gutticollis*	O, S
*Anax junius*	J, M	*Diglotta* sp.	Ch	*Hydrochara* sp.	Ch	*Micrathyria hagenii*	C, M	*Rhantus* sp.	O, S
*Antocha* sp.	Ch	*Dineutus ciliatus*	G, O	*Hydrochus* sp.	C, J, M, N, S	*Microcylloepus inaequalis*	Ch, G	*Rhionaeschna psilus*	M
*Apteraliplus* sp.	C, S	*Dineutus discolor*	Ch	*Hydroisotoma* sp.	Ch	*Microvelia beameri*	Ch	*Rhionaeschna* sp.	M, S
*Aquarius* sp*.*	J	*Dixella* sp.	Ch	*Hydrometra australis*	Ch	*Microvelia* sp.	C, Ch, J, M, N, O	*Scirtes* sp.	J
*Archilestes* sp.	M	*Dryops* sp*.*	Ch	*Hydrometra* sp.	C, Ch, N	*Neocylloepus* sp.	Ch, M	*Simulium* sp.	Ch, M
*Argia anceps*	N	*Dubiraphia* sp.	Ch	*Hydrophilus insularis*	O	*Neoperla* sp.	C, Ch, G, J, M, N, S	*Steinovelia stagnalis*	Ch
*Argia fissa*	M, N, S	*Dytiscus* sp.	J, M	*Hydrophilus smaragdinus*	O	*Nerthra mexicana*	Ch, O	*Stenelmis* sp.	Ch
*Argia oculata*	C, Ch, G, N, O	*Enallagma* sp.	C, J	*Hydrophilus* sp.	G, S	*Nerthra* sp.	C, O	*Stenus* sp.	Ch
*Argia oenea*	Ch, G, O	*Enochrus blatchleyi*	O	*Hydrophilus triangularis*	Ch	*Notomicrus* sp.	C	*Stratiomys* sp.	J, S
*Argia pulla*	C, Ch, G, M, N, O, S	*Enochrus mexicanus*	C, Ch, J, M, N, O, S	*Hydropsyche betteni*	Ch	*Notonecta* sp.	G, J, M, O, S	*Suphis* sp.	J, M, S
*Atopsyche* sp.	Ch	*Enochrus ochraceus*	O	*Hydropsyche* sp.	Ch, M	*Ochterus* sp.	Ch	*Suphisellus lineatus*	C, N, S
*Baetis* sp.	Ch	*Enochrus pseudochraceus*	M	*Hydroscapha* sp.	S	*Optioservus* sp.	Ch	*Suphisellus* sp.	J, M, N, O
*Baetodes* sp.	Ch	*Enochrus pygmaeus*	C, G, J, M, O, S	*Ischnura capreolus*	C, Ch, N	*Ordobrevia* sp.	Ch, M	*Sympetrum illotum*	M, S
*Barbaetis* sp.	Ch	*Erpetogomphus elaps*	Ch, G, N, O	*Ischnura demorsa*	C, J, M, N, O, S	*Orthemis ferruginea*	J, M, S	*Tabanus* sp.	M
*Belostoma* sp.	C, Ch, J, S	*Erpetogomphus eutainia*	Ch, G, O	*Ischnura hastata*	Ch, G, M, N, O, S	*Orthemis* sp.	M	*Telebasis salva*	C, J, M, N, S
*Berosus arneti*	S	*Erpetogomphus* sp.	Ch	*Ischnura ramburii*	C, G, J, M, N, O, S	*Pachydiplax longipennis*	C	*Telebasis* sp.	C, N, S
*Berosus exiguus*	O	*Erythemis attala*	C, M, N, O	*Ischnura* sp.	C, Ch, J, M, N, S			*Tenagobia* sp.	M
*Berosus infuscatus*	O, S	*Erythemis plebeja*	C, Ch, G, J, N, O, S	*Isonychia* sp.	Ch	*Pachydrus princeps*	G, M	*Thermonectus basillaris*	J, O, S
*Berosus mexicanus*	C, G, J, M, N, O, S	*Erythemis* sp.	M, N, S	*Laccobius* sp.	M, N	*Pachydrus* sp*.*	M	*Thermonectus marmoratus*	G, O, S
*Berosus sayi*	M	*Erythemis vesiculosa*	C, G, N, O	*Laccodytes* sp.	Ch, C, M, S	*Palaemnema desiderata*	Ch, O	*Thermonectus ornaticollis*	S
*Bibiocephala grandis*	Ch	*Erythrodiplax* sp.	C, M	*Laccophilus fasciatus*	Ch, C, G, J, M, N, O, S	*Pantala flavescens*	C, Ch, G, N, S	*Thermonectus* sp.	Ch, C, J, O, S
*Bidessonotus* sp.	C	*Erythrodiplax umbrata*	C, G, J, N, O	*Laccophilus hyalinus*	M	*Pantala hymenaea*	J, S	*Tramea abdominalis*	C, J
*Brachydeutera* sp.	S	*Gelastocoris oculatus*	J, N	*Laccophilus maculosus*	G, J, O, S	*Pantala* sp.	Ch. S	*Trepobates pictus*	N
*Brechmorhoga mendax*	N	*Gelastocoris* sp.	M, N, S	*Laccophilus pictus*	Ch, G, J, O, S	*Paracloeodes* sp.	Ch	*Trepobates* sp.	Ch, C, J, N
*Brechmorhoga praecox*	Ch, G, N, O	*Gerris* sp.	Ch	*Laccophilus* sp.	C, Ch, J, M, S	*Paracymus armatus*	Ch	*Triacanthagyna* sp.	M
*Brechmorhoga vivax*	Ch	*Gonielmis* sp.	Ch	*Laccophilus undatus*	C	*Paracymus confusus*	O	*Trichocorixa* sp.	O, S
*Bryothinusa* sp.	Ch	*Graphoderus* sp.	J	*Lara* sp.	S	*Paracymus regularis*	C, Ch, M, N, O, S	*Tricorythodes* sp.	Ch
*Buenoa* sp.	C, Ch, G, J, M, N, O, S	*Graptocorixa* sp.	J, S	*Leptobasis vacillans*	M	*Paradelphomyia* sp.	Ch	*Trochopus* sp.	Ch
*Caloparyphus greylockensis*	Ch	*Gyrinus parcus*	O	*Leptohyphes* sp.	Ch	*Paraleptophlebia* sp.	Ch	*Tropisternus blatchleyi*	S
*Camelobaetidius* sp.	Ch	*Haliplus* sp.	J, M	*Leptonema* sp.	Ch	*Pelocoris* sp.	C, Ch, G, J, M, N, O, S	*Tropisternus collaris*	C, G, J, M, N, O, S
*Celina* sp.	J	*Hebrus sobrinus*	Ch	*Lestes alacer*	M, O	*Pelonomus obscurus*	O	*Tropisternus lateralis*	J, M, O, S
*Ceratopsyche* sp.	Ch	*Helichus* sp.	Ch, S	*Lestes tenuatus*	G, J, M, N, O	*Pelonomus* sp.	C	*Tropisternus mixtus*	M, N
*Chaetarthria* sp.	M, S	*Helobata* sp.	Ch, S	*Lestes tikalus*	Ch, C, O	*Peltodytes dietrichi*	S	*Tropisternus paredesi*	S
*Chimarra* sp.	Ch	*Helochares normatus*	C, G, S	*Leucotrichia* sp.	Ch	*Peltodytes muticus*	M	*Tropisternus* sp.	C, G, J, M, N, O, S
*Copelatus caelatipennis*	O	*Helochares sallaei*	S	*Libellula foliata*	Ch	*Peltodytes* sp.	S	*Uvarus* sp.	C, M, S
*Copelatus* sp.	J, M	*Helophorus* sp.	J	*Liodessus fuscatus*	G, O, S	*Perissolestes* sp.	M	*Zaitzevia* sp.	Ch
*Corisella decolor*	M	*Hemerodromia* sp.	M	*Liodessus* sp.	C, J, M, N, S	*Perithemis* sp.	S		
*Corydalus cornutus*	C, Ch, M	*Henochrus* sp.	Ch	*Lipogomphus* sp.	Ch, C, J	*Petrophila confusalis*	Ch		

*C = Colima, Ch = Chiapas, G = Guerrero, J = Jalisco, M = Michoacán, N = Nayarit, O = Oaxaca, S = Sinaloa.

### Anopheline diversity

**Figure 5:**
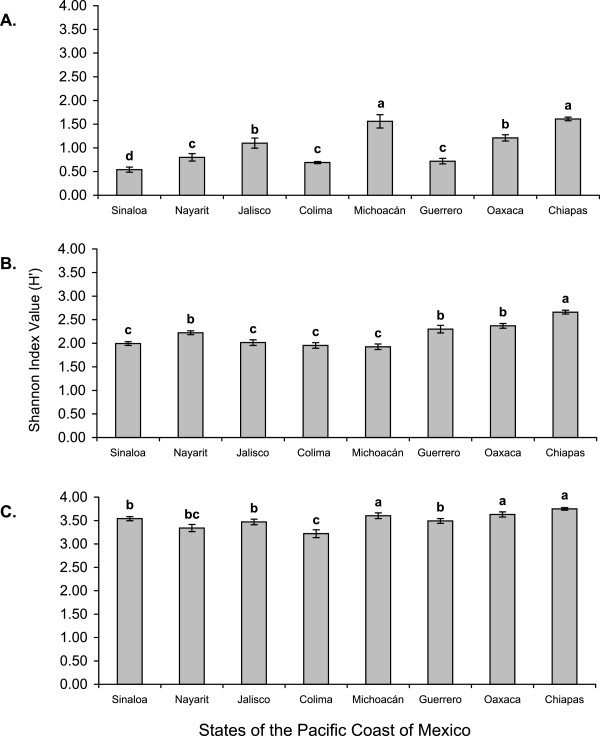
**Shannon index (H’) values for (A) anophelines, (B) culicids and (C) aquatic insects from samples taken in eight states along the Pacific coast of Mexico.** Columns labeled with different letters differ significantly for comparisons of States within each graph (*P* <0.001 pairwise randomization test).

**Figure 6:**
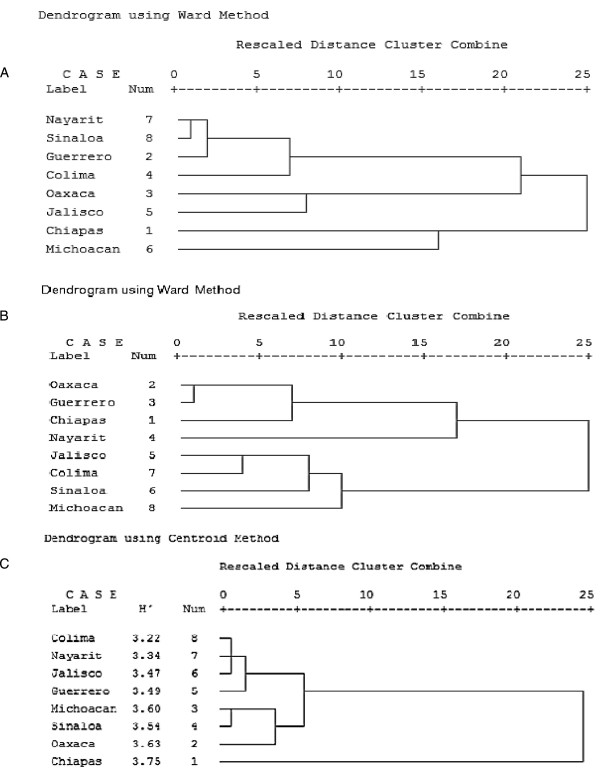
**Dendrogram generated by cluster analysis using Ward and Centroid methods for (A) anophelines, (B) culicids and (C) aquatic insects.** Samples were obtained from eight states along the Pacific coast of Mexico.

Shannon index diversity values differed between states (δ = 0.39-1.06; *P* < 0.001), and allowed states to be classified into four groups (Figure 5A). The highest diversity values were observed in collections made in the states of Chiapas and Michoacán (H’ = 1.61 and 1.56, respectively). Collections made in the states of Oaxaca and Jalisco generated index values of 1.21 and 1.10, respectively. A third group of states comprised the states of Nayarit, Colima and Guerrero with diversity values of 0.80, 0.72 and 0.69, respectively. Finally, the state of Sinaloa had the lowest H’ value at 0.54, which did not differ significantly from the value calculated for the state of Colima. Jackknifing indicated that diversity index values were underestimated by 4.14% for *Anopheles* spp. (Table 3). Diversity index findings were consistent with the cluster analysis, which also discriminated four groups depending on their species abundance, with the only difference being that in the analysis of diversity, Sinaloa clustered in a group that comprised Guerrero, Nayarit and Colima (Figure 6A).

**Table 3:** Values of total diversity estimated by jackknife for culicid, anopheline and aquatic insects sampled in eight states of the Pacific coast of Mexico

**Source**	Number of analyzed samples**	**Values of *H*** **´**	**Pseudovalues *ϕ***	**Jackknifing (± SE)**	Confidence limits (95%)*	**Error (%)**
Culicids	All (8)	2.87				
	(2,3,4,5,6,7,8)	2.72	2.81			
	(1,3,4,5,6,7,8)	2.88	1.06			
	(1,2,4,5,6,7,8)	2.88	1.48			
	(1,2,3,5,6,7,8)	2.89	1.48	3.00 ± 0.15	2.65 - 3.35	4.57
	(1,2,3,4,6,7,8)	2.88	1.48			
	(1,2,3,4,5,7,8)	2.84	1.69			
	(1,2,3,4,5,6,8)	2.82	1.27			
	(1,2,3,4,5,6,7)	2.90	1.06			
	All (8)	1.48				
Anophelines	(2,3,4,5,6,7,8)	1.29	3.92			
	(1,3,4,5,6,7,8)	1.54	2.80			
	(1,2,4,5,6,7,8)	1.48	2.80			
	(1,2,3,5,6,7,8)	1.48	2.73	1.54 ± 0.20	1.07 - 2.01	4.14
	(1,2,3,4,6,7,8)	1.48	2.80			
	(1,2,3,4,5,7,8)	1.45	3.08			
	(1,2,3,4,5,6,8)	1.51	3.22			
	(1,2,3,4,5,6,7)	1.54	2.66			
Aquatic insects	All (8)	4.46				
	(2,3,4,5,6,7,8)	4.44	4.60			
	(1,3,4,5,6,7,8)	4.45	4.53			
	(1,2,4,5,6,7,8)	4.45	4.53			
	(1,2,3,5,6,7,8)	4.45	4.53	4.61 ± 0.04	4.52 – 4.70	3.34
	(1,2,3,4,6,7,8)	4.42	4.74			
	(1,2,3,4,5,7,8)	4.46	4.46			
	(1,2,3,4,5,6,8)	4.41	4.81			
	(1,2,3,4,5,6,7)	4.43	4.67			

*Confidence intervals were calculated by bootstrap.

**The numbers 1 to 8 represent each of the states in the following order, 1. Chiapas, 2. Guerrero, 3. Oaxaca, 4. Colima, 5. Jalisco, 6. Michoacán, 7. Nayarit, 8. Sinaloa.

### Culicid diversity

Significant differences were detected between culicid diversity values from the eight states. Culicid diversity values fell into one of three groups: first, Chiapas state with a diversity index value of 2.66 that was significantly higher than the values of the seven remaining states. The second group comprised the states of Oaxaca (H’ = 2.37), Guerrero (H’ = 2.30) and Nayarit (H’ = 2.22) with similar diversity values. The third group comprised Jalisco (H’ = 2.01), Sinaloa (H’ = 1.99), Colima (H’ = 1.95) and Michoacán (H’ = 1.93) with the lowest diversity values (Figure 5B). Cluster analysis broadly supported these findings, the main difference being that Chiapas clustered with Oaxaca and Guerrero, rather than forming a separate group (Figure 6B). Jackknifing indicated that culicid diversity index values were underestimated by 4.57% (Table 3).

### Aquatic insect diversity

Diversity analyses on aquatic insect samples (Figure 5C), indicated that Chiapas had the highest Shannon index value (3.75), followed by the states of Oaxaca (3.63), Michoacan (3.60). Intermediate H’ values were estimated for the states of Sinaloa, Guerrero, Jalisco and Nayarit that varied between 3.34 and 3.54. The lowest index value was calculated for the state of Colima (3.22), which was significantly lower than all other values, except that calculated for the state of Nayarit. Jackknifing indicated that H’ values were underestimated by 3.34% for aquatic insects (Table 3). Cluster analysis supported the existence of three diversity groups consisting of Chiapas alone (Figure 6C), the second group consisting of Oaxaca, Michoacan and Sinaloa and the third group consisting of Guerrero, Jalisco, Nayarit and Colima. Minor differences in the placement of states within a particular group were observed, but the overall patterns were in agreement with the calculated diversity index values.

### Similarity analyses and correlations

Morisita-Horn similarity index values for comparison of species composition of mosquitoes among the states of Chiapas-Oaxaca or Chiapas-Guerrero were estimated at 70% or at 91% in the comparison of the states of Oaxaca-Guerrero (Table 4). Similar results were observed in the cluster analysis, in which culicids from these three states clustered in a single group (Figure 6B). Similarity in species composition of mosquitoes among the states of Jalisco, Colima and Sinaloa were close to 70%, whereas comparison of the mosquito composition of Michoacan and Jalisco was extremely high at 92% (Table 4). These findings were consistent with those of the cluster and diversity analyses that included the states of Jalisco, Colima, Sinaloa and Michoacán within a group according to their species composition or diversity (Figures 5A and 6A).

**Table 4:** Similarity values for species composition of mosquitoes from eight states of the Pacific coast of Mexico, using Morisita Horn index

	**Chiapas**	**Oaxaca**	**Guerrero**	**Nayarit**	**Jalisco**	**Sinaloa**	**Colima**	**Michoacán**
Chiapas	1.00	0.71	0.73	0.51	0.27	0.44	0.43	0.20
Oaxaca	0.71	1.00	0.91	0.53	0.48	0.52	0.50	0.32
Guerrero	0.73	0.91	1.00	0.60	0.43	0.55	0.56	0.27
Nayarit	0.51	0.53	0.60	1.00	0.26	0.51	0.27	0.16
Jalisco	0.27	0.48	0.43	0.26	1.00	0.46	0.68	0.91
Sinaloa	0.44	0.52	0.55	0.51	0.46	1.00	0.69	0.36
Colima	0.43	0.50	0.56	0.27	0.68	0.69	1.00	0.57
Michoacán	0.20	0.32	0.27	0.16	0.91	0.36	0.57	1.00

The presence of mosquito larvae was not significantly correlated with the prevalence of aquatic insect predators of the orders Coleoptera (Spearman’s *r*_*s*_ = -0.056, N = 180), or Hemiptera (Spearman’s *r*_*s*_ = -0.141, N = 80). Additional correlations were not performed as the low numbers of independent points reduced the validity of the correlation, e.g., as was the case for Odonata.

## Discussion

The diversity of mosquito species was determined for each of eight states along 2,500 km of the Pacific coast of Mexico. This represents the most complete mosquito survey of this region to date and markedly expands the information provided by the only previous detailed studies on the mosquito fauna of Mexico performed in the 1950’s [28,29]. A large and taxonomically diverse group of aquatic insects associated with mosquito oviposition sites was also identified.

The selection of sampling sites within each state was based on established information available on the type of ecosystem and habitat used by anophelines and culicids as likely oviposition sites. Decisions on which habitats to sample, and in which period of the year, doubtless affected the likelihood of identifying particular species. This is because the oviposition site preferences of each mosquito species reflect a characteristic and habitat-specific suite of biotic and abiotic factors that favor the development and survival of their offspring [9]. However, such targeted sampling regimes are an inevitable consequence of finite financial and human resources available for faunistic studies of this kind.

Globally, the family Culicidae comprises 3,525 species distributed among 111 genera [49]. Mosquito diversity tends to be highest in tropical habitats [4]. In the present study a total of 15 genera and 74 species were identified, which represents ~2% of global species. Within Mexico, the recorded mosquito fauna consists of between 15 and 16 genera and between 217 and 239 species [5,6], although the genera *Chagasia*, *Orthopodomyia*, *Shannoniana*, and *Toxorhynchites* were excluded from these reports because these genera have no species of medical importance. The mosquito species reported in the present study represented 30 - 34% of the total Mexican mosquito fauna. Surveys on the mosquito fauna of Mexico are remarkably sparse. A total of 52 species from 11 genera have been recorded for the state of Guerrero State [17,28,29], in addition to species from other genera, such as *Toxorhynchites* species, that were not considered in the present study. This compared with 38 species from 10 genera recorded in the present study. In the remaining seven states 17–54 species were identified from 7–14 genera with the highest taxa richness in the state of Chiapas and the lowest in Nayarit. Overall, the present study extended the number of species records by just two species in Guerrero state and by up to 14 species in Chiapas state (Table 1). Similarly, the percentage of recorded species was increased by just 4% in Guerrero (2 additional species/52 previously reported species), but was almost doubled for Colima (9 new records/10 reported species), underlining the value of the present study to understanding the distribution of vector species in this region. Surveys have also been conducted in other states of Mexico, such as Veracruz [7], Yucatán [50-52], and Quintana Roo [53] but their geographical location, ~1000 km from the Pacific coast of Mexico, and marked differences in the type of ecosystems present in the Atlantic and Caribbean coasts compared to the Pacific coast, means that these studies are of limited relevance to the present findings.

The diversity index values of mosquitoes in the eight states included in this study ranged from 1.93 for the state of Michoacan to 2.66 for the state of Chiapas. There are no previous systematic surveys of mosquitoes in the Pacific coast region, with the exception of the studies performed in the 1950’s [28,29], and one study in Guerrero in the 1970’s [17]. The high mosquito diversity present in the Pacific coast region is likely due to a combination of the overlap between Neartic and Neotropical fauna and the great diversity of ecosystems present in this region, which is largely responsible for the status of Mexico as one of the world’s megadiverse countries. In this respect, the states of Chiapas and Oaxaca represent the states with the highest diversity in Mexico, followed by the states of Veracruz, Guerrero and Michoacán [54].

Globally, 465 species of *Anopheles* have been recognized, of which approximately 70 species have the capacity to transmit human malaria parasites [9,55], and 41 are considered to be dominant vector species, capable of transmitting malaria at a level of major concern to public health [56]. Approximately one third of the population of Mexico lives in areas prone to malaria transmission [57]. Between 26 and 28 species of *Anopheles* have been reported in Mexico [5,6,58], but only two of these are considered as being of major public health concern: *An. pseudopunctipennis* and *An. albimanus* are the principal vectors of *P. vivax* in mountain foothills and coastal areas, respectively [13,59].

A total of 18 species of anophelines were recorded during this study, in addition to one previous species record (*Anopheles xelajuensis*) [28]. The state of Chiapas had the highest anopheline species richness (15) and the highest diversity value (1.61), followed by the states of Michoacan (9 species) and Oaxaca (9 species). Our study expands the anopheline fauna of seven of the eight states studied (Table 1). Specifically, *An. crucians* is a new species record for Chiapas, Oaxaca and Colima states; *An. franciscanus* is a new record for Chiapas, Oaxaca and Jalisco states; *An. freeborni* is a new record for Michoacán state; *An. punctimacula* is a new record for Sinaola state, and *An. punctipennis* is a new record for Jalisco and Nayarit states. No new anopheline records were obtained for the state of Guerrero [17].

The mosquitoes *Ae. aegypti* and *Ae. albopictus* are vectors of dengue in tropical and subtropical regions throughout the world and represent a major public health concern [60]. However, despite its dramatic global expansion in the last three decades, *Ae. albopictus* is believed to have a lesser role in dengue virus transmission compared to *Ae. aegypti*, due to differences in host preference and vector competence [61]. Of these, only *Ae. aegypti* was present in all eight states of our study area, whereas the exotic invasive species *Ae. albopictus* was only present in Chiapas. In Mexico, *Ae. albopictus* was first recorded in 1988 in the northeastern state of Tamaulipas [62,63], but was also reported in the southern state of Chiapas in 2002 [64], and recently in the central state of Morelos [65] and the Gulf coast states of Yucatán [66], and Veracruz [67]. Given the presence of *Ae. albopictus* in Chiapas, its introduction, establishment, and spread to the other states of the Pacific coast is an issue of considerable concern. As invasive mosquitoes have the potential for biotic impacts on native species, ecosystems, and on human and animal health [68], an invasive mosquito that replaces a resident species via competition may alter disease transmission and amplify the importance of vector borne disease in affected areas [69].

The taxa richness and diversity of aquatic insects associated with breeding sites of mosquitoes in this study was high, with a total of 10 orders, 57 families, 166 genera and 247 species, with Shannon index values between 3.22 and 3.75, depending on the state in question. The orders of greatest abundance and species richness were Coleoptera, Odonata and Hemiptera that represented 87% of the total species sampled.

In northern Mexico, 39 genera, 27 families, and 7 orders of aquatic insects were reported in association with oviposition sites of *An. pseudopunctipennis*[18]. Similarly, 52 genera, 19 families, and 3 orders of aquatic insects were reported in association with *An. albimanus* oviposition sites in southern Chiapas, of which Coleoptera was the most abundant and diverse order [19]. In comparison, another study in southern Chiapas reported 90 genera, 40 families and 10 orders of aquatic insects associated with oviposition sites of *An. pseudopunctipennis.* In this case Shannon index values fluctuated between 2.4 and 3.2 [21]. However, these previous studies were focused on the oviposition sites of single species of mosquitoes, and restricted to a single state, which contrasts with our effort to evaluate the diversity of aquatic insects associated with immature mosquitoes along the Pacific coast of Mexico. Comparable studies in temperate regions have reported markedly lower diversity of aquatic insects, which was ascribed to the harsh environmental conditions and instability of the habitat studied [11].

The presence of mosquito larvae was not significantly correlated with the abundance of aquatic insect predators of the orders Coleoptera and Hemiptera. A negative tendency was observed in the correlation between mosquito abundance and predator abundance, but in neither case was this significant, probably due to the large number of observations of single individuals in the dataset used. Previous studies have highlighted the importance of these orders, since many members of these orders are known to prey on mosquito larvae [26,70]. In addition to regulating mosquito populations by direct predation, a number of these predators can influence adult mosquito oviposition decisions or affect the rate of development of immature stages [10,71-73]. There is renewed interest in employing natural enemies for the control of mosquito larvae to complement existing vector control measures. In this respect, due to their numerical and functional responses, naturally occurring predators can be a significant density dependent mortality factor in the regulation of mosquito populations [74,75]. However, apart from some species of fish and bacteria-based biological insecticides, the adoption of biological vector control in most countries remains extremely limited.

## Conclusion

This study represents the first systematic update to the inventory and distribution of mosquitoes in Mexico in over five decades. The majority of the individuals reported were catalogued in databases of mosquitoes and aquatic insects in Mexico’s National Biodiversity Information System (SNIB-CONABIO), and are available for public inspection. We believe this represents a valuable contribution to recording the diversity and geographic distribution of the mosquitoes and aquatic insects in this region that is affected by major vector borne diseases, particularly dengue and malaria. Numerous new species records for different states along the Pacific coast are reported. Considerably greater sampling effort would be required to yield realistic estimates of total mosquito species richness of the country, particularly for the Mexican Culicidae inventory, given the great diversity of ecosystems present in this megadiverse country.

## Competing interests

The authors declare that they have no competing interests.

## Authors’ contributions

MCM obtained funding via a competitive proposal. MCM, JGB, CFM, AU, AOB, MM performed field studies. JGB, MCM, CFM, AU, MM performed mosquito rearing. AOB identified mosquitoes. JGB, HQM, RNG identified aquatic insects. JGB, TW performed statistical analyses. JGB, CFM, TW wrote the manuscript. All authors read and approved the final version of the manuscript.

## Supplementary Material

Additional file 1: Table S1 Numbers of field-collected mosquitoes that were reared in the laboratory and identified to species following adult emergence. Columns indicate numbers of each species in samples taken from eight states of the Pacific coast of Mexico. Click here for file

## References

[B1] Campbell-Lendrum D Molyneux D Amerasinghe F Epstein P, Githeko A, Rabinovich J, Weinstein P Ecosystems and vector-borne disease control Ecosystems and Human Well-Being. Findings of the responses working group of the Millennium Ecosystem Assessment. Volume 3 2005 Washington DC: Island Press 353 372

[B2] Townson H Nathan MB Zaim M Guillet P Manga L Bos R Kindhauser M Exploiting the potential of vector control for disease prevention Bull WHO 2005 83 942 947 16462987 PMC2626501

[B3] WHO International Statistical Classification of Diseases and Related Health Problems 10th Revision (ICD-10) 2010 http://apps.who.int/classifications/icd10/browse/2010/en#/B74

[B4] Harbach RE The Culicidae (Diptera): A review of taxonomy, classification and phylogeny Zootaxa 2007 1668 591 638

[B5] Darsie RF Jr A survey and bibliography of the mosquito fauna of Mexico (Diptera: Culicidae) J Am Mosq Contr Assoc 1996 12 298 306 8827608

[B6] Ibáñez-Bernal S Strickman S Martínez-Campos C Llorente BJ, García AN, González E Culicidae (Diptera) Biodiversidad, Taxonomía y Biogeografía de Artrópodos de México: Hacia una Síntesis de Su conocimiento 1996 Mexico City: Universidad Nacional Autónoma de México 591 602

[B7] Beltrán-Aguilar A Ibáñez-Bernal S Mendoza-Palmero F Sandoval-Ruiz CA Hernández-Xoliot RA Taxonomía y distribución de los anofelinos en el Estado de Veracruz, México (Diptera: Culicidae, Anophelinae) Acta Zool Mex 2011 27 601 755

[B8] Knight KL Stone A A Catalog of the Mosquitoes of the World (Diptera: Culicidae). Volume 6 1977 The Thomas Say Foundation, Langham, Maryland: Entomological Society of America

[B9] Service MW Medical Entomology for Students 2004 3 Cambridge, UK: Cambridge University Press

[B10] Juliano SA Species interactions among larval mosquitoes: context dependence across habitat gradients Ann Rev Entomol 2009 54 37 56 10.1146/annurev.ento.54.110807.090611 19067629 PMC2664081

[B11] Rochlin I Dempsey ME Iwanejko T Ninivaggi DV Aquatic insects of New York salt marsh associated with mosquito larval habitat and their potential utility as bioindicators J Ins Sci 2011 11 172 10.1673/031.011.17201 PMC3463129 22957707

[B12] Savage HM Rejmankova E Arredondo-Jiménez JI Roberts DR Rodríguez MH Limnological and botanical characterization of larval habitats for two primary malarial vectors, *Anopheles pseudopunctipennis* and *Anopheles albimanus*, in coastal areas of Chiapas State, Mexico J Am Mosq Contr Assoc 1990 6 612 620 2098467

[B13] Fernández-Salas I Rodríguez MH Roberts DR Rodríguez MC Wirtz RA Bionomics of adult *Anopheles pseudopunctipennis* (Diptera: Culicidae) in the Tapachula foothills area of Southern Mexico J Med Entomol 1994 3 663 670 7966168 10.1093/jmedent/31.5.663

[B14] Manguin S Roberts DR Peyton EL Rejmankova E Pecor J Characterization of *Anopheles pseudopunctipennis* larval habitats J Am Mosq Contr Assoc 1996 12 619 626 9046466

[B15] Rubio-Palis Y Zimmerman RH Ecoregional classification of malaria vectors in the neotropics J Med Entomol 1997 34 499 510 9379453 10.1093/jmedent/34.5.499

[B16] Sinka ME Rubio-Palis Y Manguin S Patil AP Temperley WH Boeckel TV Kabaria CW Harbach RE Hay S Gething PW The dominant *Anopheles* vectors of human malaria in the Americas: occurrence data, distribution maps and bionomic précis Parasit Vectors 2010 3 117 10.1186/1756-3305-3-117 20712879 PMC2936890

[B17] García-Aldrete AN Pletsch DJ Fauna de mosquitos (Diptera: Culicidae), en la zona turística de Ixtapa, Guerrero Folia Entomol Mex 1976 34 71 83

[B18] Delgado-Gallardo ML Badii MH Quiroz-Martinez H Diversidad ecológica de las comunidades acuáticas cohabitando con *Anopheles pseudopunctipennis* (Diptera: Culicidae) en el arroyo la Ciudadela, en el municipio de Benito Juárez, Nuevo León, México Southwest Entomol 1994 19 77 81

[B19] Danis-Lozano R Rodríguez MH Arredondo-Jiménez JI Hernández-Ávila M Mallorca C Aquatic insects associated with *Anopheles albimanus* (Diptera: Culicidae) breeding sites in southern Mexico Env Entomol 1997 26 826 838

[B20] Bond JG Novelo-Gutiérrez R Ulloa A Rojas JC Quiroz-Martínez H Williams T Diversity, abundance, and disturbance response of Odonata associated with breeding sites of *Anopheles pseudopunctipennis* (Diptera: Culicidae) in southern Mexico Env Entomol 2006 35 1561 1568 10.1603/0046-225X(2006)35[1561:DAADRO]2.0.CO;2

[B21] Bond JG Quiroz-Martinez H Rojas JC Valle J Ulloa A Williams T Impact of environmental manipulation for *Anopheles pseudopunctipennis* Theobald control on aquatic insect communities in southern Mexico J Vect Ecol 2007 32 41 53 10.3376/1081-1710(2007)32[41:IOEMFA]2.0.CO;2 17633425

[B22] Merritt R Cummins K Berg M An introduction to the aquatic insects of North America 2008 4 Dubuque, Iowa: Kendall Hunt Publishing Co

[B23] Ward JV Aquatic Insect Ecology. Volume 1. Biology and Habitat 1992 New York: John Wiley & Sons

[B24] Malmqvist B Aquatic invertebrates in riverine landscapes Freshwat Biol 2002 47 679 694 10.1046/j.1365-2427.2002.00895.x

[B25] Rosenberg DM Resh RH Freshwater Biomonitoring and Benthic Macroinvertebrates 1993 New York: Chapman & Hall

[B26] Shaalan EA Canyon DV Aquatic insect predators and mosquito control Trop Biomed 2009 26 223 261 20237438

[B27] Instituto Nacional de Estadística Geografía e Informática Anuario Estadístico de los Estados Unidos Mexicanos 2000 Mexico City: Instituto Nacional de Estadística Geografía e Informática

[B28] Vargas L Martínez-Palacios A Anofelinos Mexicanos, Taxonomía y Distribución 1956 Mexico City: Comisión Nacional para la Erradicación del Paludismo: Secretaría de Salubridad y Asistencia

[B29] Vargas L Especies y distribución de mosquitos mexicanos no anofelinos Rev Inst Salubr Enferm Trop 1956 16 19 36 13370988

[B30] Comité Nacional para la Vigilancia Epidemiológica Aviso epidemiológico Virus del Oeste del Nilo: Incremento de casos de infección por Virus del Oeste del Nilo en los Estados Unidos de América 2012 CoNaVe/05/VIRUS DEL OESTE DEL NILO. http://www.facmed.unam.mx/deptos/salud/vigilanciaepidem/alerta_20ago2012.pdf

[B31] Darsie RF Jr Ward RA Identification and Geographical Distribution of the Mosquitoes of North America, North of Mexico 2005 2 Gainesville: University of Florida

[B32] Clark-Gill S Darsie RF Jr The mosquitoes of Guatemala, their identification, distribution and bionomics, with keys to adult females and larvae Mosq Systemat 1983 15 151 284

[B33] Wilkerson RC Strickman D Fernández-Salas I Ibáñez-Bernal S Liwak TR Clave ilustrada para la identificación de las hembras de mosquitos anofelinos de México y América Central 1993 Mexico City: Secretaría de Salud. Centro de Investigación de Paludismo

[B34] Pollard JE Investigator differences associated with a kicking method for sampling macroinvertebrates J Freshwat Ecol 1981 1 215 224 10.1080/02705060.1981.9664033

[B35] González-Soriano E Novelo-Gutiérrez R Llorente BJ, García AAN, González SE Odonata Biodiversidad, taxonomía y biogeografía de artrópodos de México: Hacia una síntesis de su conocimiento 1996 Mexico City: Universidad Autónoma de México 147 167

[B36] Westfall MJ Jr May ML Damselflies of North America 1996 Gainesville: Scientific Publishers

[B37] Novelo-Gutiérrez R Clave para la separación de familias y géneros de las náyades de Odonata de México. Parte I. Zygoptera Dugesiana 1997 4 1 10

[B38] Novelo-Gutiérrez R Clave para la identificación de familias y géneros de las náyades de Odonata de México. Parte II. Anisoptera Dugesiana 1997 4 31 40

[B39] Contreras-Ramos A List of species of Neotropical Megaloptera (Neuropterida) Proc Entomol Soc Wash 1999 101 273 284

[B40] Needham JG Westfall MJL May M Dragonflies of North America 2000 Gainesville: Scientific Publishers

[B41] Epler JH Identification Manual for the Aquatic and Semi-aquatic Heteroptera of Florida (Belostomatidae, Corixidae, Gelastocoridae, Gerridae, Hebridae, Hydrometridae, Mesoveliidae, Naucoridae, Nepidae, Notonectidae, Ochteridae, Pleidae, Saldidae, Veliidae) 2006 http://bugguide.net/node/view/368765

[B42] Epler JH The Water Beetles of Florida - an Identification Manual for the families Chrysomelidae, Curculionidae, Dryopidae, Dytiscidae, Elmidae, Gyrinidae, Haliplidae, Helophoridae, Hydraenidae, Hydrochidae, Hydrophilidae, Noteridae, Psephenidae, Ptilodactylidae and Scirtidae 2010 http://publicfiles.dep.state.fl.us/dear/labs/biology/biokeys/beetles10.pdf

[B43] Comisión Nacional para el Conocimiento y Uso de la Biodiversidad (CONABIO) Sistema de Información Biótica 4.5. Manual de Usuario 2006 Mexico City: Fideicomiso Fondo para la Biodiversidad

[B44] Magurran AE Measuring Biological Diversity 2004 Oxford, UK: Blackwell

[B45] Henderson PA Seaby RM Species Diversity and Richness III v 3.0.2 2002 Lymington, UK: Pisces Conservation Ltd

[B46] Solow AR A simple test for change in community structure J Anim Ecol 1993 62 191 193 10.2307/5493

[B47] Southwood TRE Henderson PA Ecological Methods 2000 3 Oxford, UK: Blackwell

[B48] Wolda H Similarity indices, sample size and diversity Oecologia 1981 50 296 302 10.1007/BF00344966 28309044

[B49] Harbach RE Culicidae (Diptera). Mosquito Taxonomic Inventory 2013 http://mosquito-taxonomic-inventory.info/valid-species-list

[B50] Nájera-Vázquez R Dzul F Sabido M Tun-Ku E Manrique-Saide P New distribution records of mosquitoes (Diptera: Culicidae) for Yucatan, Mexico Entomol News 2004 115 181 190

[B51] Zapata-Peniche A Manrique-Saide P Rebollar-Téllez EA Che-Mendoza A Dzul-Manzanilla F Identificación de larvas de mosquitos (Diptera: Culicidae) de Mérida, Yucatán, México y sus principales criaderos Rev Biomed 2007 18 3 17

[B52] Dzul MF Manrique SP Che MA Rebollar TE Durán R, Méndez M Mosquitos de Yucatán Biodiversidad y Desarrollo Humano en Yucatán 2010 Mérdida, México: CICY, PPD-FMAM, CONABIO, SEDUMA http://www.seduma.yucatan.gob.mx/biodiversidad-yucatan/libro-biodiversidad-yucatan.php

[B53] Ortega MAI Avila PM Elizondo-Quiroga A Harbach RE Quetzaly K Siller-Rodríguez QK Fernández-Salas I The mosquitoes of Quintana Roo State, Mexico (Diptera: Culicidae) Acta Zool Mex 2010 26 33 46

[B54] Mittermeier RA Goesttsch C Sarukhán J, Dirzo R La importancia de la diversidad biológica de México México ante los retos de la biodiversidad 1992 Mexico: Mexico City: CONABIO

[B55] Service MW Townson H Gilles HM, Warrell DA The *Anopheles* vector Essential Malariology 2002 4 London: Arnold 59 84

[B56] Sinka ME Bangs MJ Manguin S Rubio-Palis Y Chareonviriyaphap T Coetzee M Mbogo CM Hemingway J Patil AP Temperley WH Gething PW Kabaria CW Burkot TR Harbach RE Hay SI A global map of dominant malaria vectors Parasit Vectors 2012 5 69 10.1186/1756-3305-5-69 22475528 PMC3349467

[B57] Rodríguez MH Hernández-Ávila JE Betanzos-Reyes AF Danis-Lozano R González-Cerón L Durán-Arenas LG Méndez-Galván JF Vázquez-Mellado RM Velásquez-Monroy OJ Holguín-Bernal Tapia-Coyner R An ecosystem approach study of malaria transmission and control interventions in southern Mexico. Forum 8 2004 Mexico City: Global Forum for Health Research http://bvs.per.paho.org/texcom/cd048449/rodrigue.pdf

[B58] Gaffigan TV Wilkerson RC Pecor JE Stoffer JA Anderson T Systematic Catalog of Culicidae. Silver Spring, Maryland: Walter Reed Army Institute of Research. Walter Reed Biosystematics Unit: Division of Entomology http://www.mosquitocatalog.org/default.aspx?pgID=2

[B59] Rodriguez MH Loyola EG Sparks NV Malaria in Mexico Proceedings and Papers of the 58th Annual Conference of the California Mosquito and Vector Control Association, 28–31 January 1990 1990 Sacramento, CA: CMVCA Press 49 52

[B60] Brady OJ Johansson MA Guerra CA Bhatt S Golding N Pigott DM Delatte H Grech MG Leisnham PT Maciel-de-Freitas R Styer LM Smith DL Scott TW Gething PW Hay SI Modelling adult *Aedes aegypti* and *Aedes albopictus* survival at different temperatures in laboratory and field settings Parasit Vectors 2013 6 351 10.1186/1756-3305-6-351 24330720 PMC3867219

[B61] Lambrechts L Scott TW Gubler DJ Consequences of the expanding global distribution of *Aedes albopictus* for dengue virus transmission PLoS Negl Trop Dis 2010 4 1 10 10.1371/journal.pntd.0000646 PMC2876112 20520794

[B62] Womack ML Distribution, abundance and bionomics of *Aedes albopictus* in southern Texas J Am Mosq Contr Assoc 1993 9 367 369 8245952

[B63] Ibáñez-Bernal S Martínez-Campos C *Aedes albopictus* in Mexico J Am Mosq Contr Assoc 1994 10 231 232 8965073

[B64] Casas-Martínez M Torres-Estrada JL First evidence of *Aedes albopictus* (Skute) in Southern Chiapas, Mexico Emerg Infect Dis 2003 9 606 607 10.3201/eid0905.020678 12737750 PMC2972768

[B65] Villegas-Trejo A Manrique-Saide P Che-Mendoza A Cruz-Canto W Fernandez MG González-Acosta C Dzul-Manzanilla F Huerta H Arredondo-Jiménez JI First report of *Aedes albopictus* and other mosquito species in Morelos, Mexico J Am Mosq Contr Assoc 2010 26 321 323 10.2987/10-6014.1 21033059

[B66] Salomón-Grajales J Lugo-Moguel GV Tinal-Gordillo VR de la Cruz-Velázquez J Beaty BJ Eisen L Lozano-Fuentes S Moore CG García-Rejón JE *Aedes albopictus* mosquitoes, Yucatan peninsula, Mexico Emerg Infect Dis 2012 18 525 527 10.3201/eid1803.111626 22377491 PMC3309596

[B67] de la Cruz-Francisco V Veda-Moreno DI Valdés-Murillo A Ecological aspects of larval incidence of mosquitoes (Diptera: Culicidae) in Tuxpan, Veracruz, Mexico Rev Colomb Entomol 2012 38 128 133

[B68] Lounibos LP Invasions by insect vectors of human disease Annu Rev Entomol 2002 47 233 266 10.1146/annurev.ento.47.091201.145206 11729075

[B69] Juliano SA Lounibos LP Ecology of invasive mosquitoes: effects on resident species and on human health Ecol Lett 2005 8 558 574 10.1111/j.1461-0248.2005.00755.x 17637849 PMC1920178

[B70] Culler LE Lamp WO Selective predation by larval *Agabus* (Coleoptera: Dytiscidae) on mosquitoes: support for conservation based mosquito suppression in constructed wetlands Freshwat Biol 2009 54 2003 2014 10.1111/j.1365-2427.2009.02230.x

[B71] Kumar R Hwang J Larvicidal efficiency of aquatic predators: a perspective for mosquito biocontrol Zool Stud 2006 45 447 466

[B72] Legner EF Biological control of Diptera of medical and veterinary importance J Vect Ecol 1994 20 59 120

[B73] Knight TM Chase JM Goss JM Knight JJ Effects of interspecific competition, predation, and their interaction on survival and development time of immature *Anopheles quadrimaculatus* J Vect Ecol 2004 29 277 284 15707287

[B74] Kweka EJ Zhou G Gilbreath TM III Afrane Y Nyindo M Githeko AK Yan G Predation efficiency of *Anopheles gambiae* larvae by aquatic predators in western Kenya highlands Parasit Vectors 2011 4 128 10.1186/1756-3305-4-128 21729269 PMC3141748

[B75] Mereta ST Yewhalaw D Boets P Ahmed A Duchateau L Speybroeck N Vanwambeke SO Legesse W De Meester L Goethals PLM Physico-chemical and biological characterization of anopheline mosquito larval habitats (Diptera: Culicidae): implications for malaria control Parasit Vectors 2013 6 320 10.1186/1756-3305-6-320 24499518 PMC4029358

