# Supplementary material for: Diversity of mosquitoes and the aquatic insects associated with their oviposition sites along the Pacific coast of Mexico
Source: Parasit Vectors. 2014 Jan 22;7:41. doi: 10.1186/1756-3305-7-41 (PMC3923424; doi:10.1186/1756-3305-7-41)
Supplement: Additional file 1: Table S1 — Numbers of field-collected mosquitoes that were reared in the laboratory and identified to species following adult emergence. Columns indicate numbers of each species in samples taken from eight states of the Pacific coast of Mexico. [file 1756-3305-7-41-S1.doc]

**Supplemental material. Table S1: Numbers of field-collected mosquitoes that were reared in the laboratory and identified to species following adult emergence.**

Columns indicate numbers of each species in samples taken from eight states of the Pacific coast of Mexico.
